# Impairment of PGC-1 Alpha Up-Regulation Enhances Nitrosative Stress in the Liver during Acute Pancreatitis in Obese Mice

**DOI:** 10.3390/antiox9090887

**Published:** 2020-09-19

**Authors:** Sergio Rius-Pérez, Isabel Torres-Cuevas, María Monsalve, Francisco J. Miranda, Salvador Pérez

**Affiliations:** 1 Department of Physiology, Faculty of Pharmacy, University of Valencia, Avda. Vicente Andres Estelles s/n, 46100 Burjassot, Spain; sergio.rius@uv.es (S.R.-P.); Francisco.J.Miranda@uv.es (F.J.M.); 2 Neonatal Research Group, Health Research Institute La Fe, 46026 Valencia, Spain; Maria.I.Torres@uv.es; 3 Instituto de Investigaciones Biomédicas “Alberto Sols” (CSIC-UAM), Arturo Duperier, 4, 28029 Madrid, Spain; mpmonsalve@iib.uam.es

**Keywords:** acute pancreatitis, obesity, nitrosative stress, PGC-1α, liver

## Abstract

Acute pancreatitis is an inflammatory process of the pancreatic tissue that often leads to distant organ dysfunction. Although liver injury is uncommon in acute pancreatitis, obesity is a risk factor for the development of hepatic complications. The aim of this work was to evaluate the role of PGC-1α in inflammatory response regulation in the liver and its contribution to the detrimental effect of obesity on the liver during acute pancreatitis. For this purpose, we induced acute pancreatitis by cerulein in not only wild-type (WT) and PGC-1α knockout (KO) mice, but also in lean and obese mice. PGC-1α levels were up-regulated in the mice livers with pancreatitis. The increased PGC-1α levels were bound to p65 to restrain its transcriptional activity toward *Nos2*. Lack of PGC-1α favored the assembly of the p65/phospho-STAT3 complex, which promoted *Nos2* expression during acute pancreatitis. The increased transcript *Nos2* levels and the pro-oxidant liver status caused by the down-regulated expression of the PGC-1α-dependent antioxidant genes enhanced nitrosative stress and decreased energy charge in the livers of the PGC-1α KO mice with pancreatitis. It is noteworthy that the PGC-1α levels lowered in the obese mice livers, which increased the *Nos2* mRNA expression and protein nitration levels and decreased energy charge during pancreatitis. In conclusion, obesity impairs PGC-1α up-regulation in the liver to cause nitrosative stress during acute pancreatitis.

## 1. Introduction

Acute pancreatitis (AP) is an inflammatory disorder of the pancreas that often leads to a systemic inflammatory response and organ failure [1]. Currently, AP is the main cause of hospital admission for gastrointestinal problems in the USA [2], with a mortality rate of around 30% in patients who develop organ failure [3]. Several pieces of evidence suggest that oxidative and nitrosative stresses play an essential role in AP pathogenesis [4]. Oxidative stress amplifies the inflammatory process that leads to oxidative damage and contributes to the progression of extrapancreatic complications [5,6]. Nitrosative stress is a well-known feature of AP, with inducible nitric oxide synthase (NOS2) being the main source of nitric oxide (NO) in the pancreas during the course of this pathology [7,8,9,10]. In fact, *Nos2*-deficient mice exhibit a low degree of pancreatic inflammation and tissue damage in the pancreas with AP [10].

Obesity is a chronic inflammatory condition that increases the appearance of local and systemic complications and mortality in patients with AP [11,12,13]. Numerous factors contribute to systemic injury in obese patients with AP, such as the uncontrolled cytokine response, the release of unsaturated fatty acids and damage-associated molecular patterns [13]. The pulmonary, cardiovascular, and renal systems are more frequently affected in AP via these mediators [14]. On the contrary, liver damage is less common, but interestingly its appearance is used as a prognostic value in human AP and its failure invariably leads to death [15]. Remarkably, obesity only triggers hepatic injury during AP in genetically obese fa/fa Zucker rats compared to lean rats [16]. Furthermore, fatty livers can imply much higher rates for local complications, organ failure and mortality during AP [17].

PPARγ co-activator 1α (PGC-1α) is a transcriptional co-activator that is dysregulated in obesity and is important for maintenance of balance in the production of reactive oxygen species (ROS) during inflammatory processes [18]. Indeed, PGC1α modulates the expression of mitochondrial antioxidant defense genes, including manganese superoxide dismutase (*Sod2*), peroxiredoxin (*Prx*) 3, *Prx5* and catalase [19,20]. PGC-1α overexpression is known to suppress the expression of the pro-inflammatory cytokines triggered by tumor necrosis factor-α (TNF-α) in C2C12 muscle cells [21]. Low PGC-1α levels in inflamed tissues increase ROS production and contribute to increased inflammatory response [22]. 

In the present work, we address the role of PGC-1α in inflammatory response regulation in the liver during acute pancreatitis. Furthermore, we explore the precise contribution of PGC-1α in the liver to the detrimental effect of obesity on acute pancreatitis.

## 2. Materials and Methods

### 2.1. Animals

C57BL/6 J PGC-1α^−/−^ mice were originally provided by Dr. Bruce Spiegelman (Dana–Farber Cancer Institute, Harvard Medical School, Boston, MA, USA). Subsequently, a colony was established at the Institute of Biomedical Research “Alberto Sols” (Madrid, Spain) animal facility. The generation and phenotype of PGC-1α knockout (KO) mice have been described previously [23].

Male C57BL/6 J PGC-1α^+/+^ (22.4 ± 1.5 g; *n* = 12) and C57BL/6 J PGC-1α^−/−^ (21.9 ± 1.7 g; *n* = 12) mice were used and fed a standard diet. The male C57BL/6 J mice purchased from Jackson Laboratory (Bar Harbor, ME, USA) were used, and fed either standard chow (TD.08485, Envigo, Barcelona, Spain) (lean: 22.9 ± 1.0 g; *n* = 10) or a high-fat diet with 40% calories from fat (TD.88137, Envigo, Barcelona, Spain) (obese: 29.7 ± 1.8 g; *n* = 10) for 12 weeks.

All the animals were housed under standard environmental conditions (20–22 °C, 50 ± 10% humidity, 12 h light–dark cycle) with food and water ad libitum. Experiments were conducted in compliance with legislation on the protection of animals used for scientific purposes in Spain (RD 53/2013) and with EU (Directive 2010/63/EU). Protocols were approved by the Ethics Committee of Animal Experimentation and Welfare of the University of Valencia (Ethical Protocol Code A1529666350463, Valencia, Spain). This was approved by the Regional Ministry of Agriculture, Environment, Climate Change and Rural Development of Generalitat Valenciana with Code 2018/VSC/PEA/0190 type 2.

### 2.2. Experimental Model of Acute Pancreatitis

Acute pancreatitis was induced in 12-week-old mice by seven intraperitoneal cerulein injections (Sigma-Aldrich, St. Louis, MO, USA) (50 μg/kg body weight) at 1-h intervals [24]. Physiological saline (0.9% NaCl) was administered to the control group (sham mice). Animals were sacrificed 1 h after the seventh cerulein injection. Mice were sacrificed by euthanization under anesthesia with isoflurane 3–5% and were then exsanguinated. The pancreas and liver were immediately removed. Sacrifice was confirmed by cervical dislocation.

### 2.3. RNA Extraction and RT-qPCR Analysis of Gene Expression

Total RNA was isolated using TRIzol reagent (Sigma-Aldrich, St. Louis, MO, USA) following the manufacturer’s instructions. The RNA concentration was measured in a NanoDrop Lite spectrophotometer (Thermo Scientific, Waltham, MA, USA), and purity was determined by the optical density (OD) 260/280 ratio. RNA was reverse transcribed to cDNA with the PrimeScript RT Reagent Kit (Perfect Real Time) (Takara Bio Inc., Kusatsu, Japan) following the manufacturer’s instructions. The RNA levels of the genes were performed in a thermal cycler (I-Cycler + IQ Multicolor Real-Time OCR Detection System, Biorad, Hercules, CA, USA) by using the SYBR Green PCR Master Mix (Takara Bio Inc., Kusatsu, Japan). The employed specific primers are shown in Table 1.

RT-qPCR was performed by running TaqMan gene expression assays and the TaqMans PCR Master Mix (Applied Biosystems, Life Technologies Corporation, Carlsbad, CA, USA). A list of the analyzed genes and TaqMan probes is presented in Table 2.

The results were normalized using the TATA binding protein (*Tbp*) as housekeeping. The threshold cycle (CT) was determined and the relative gene expression was expressed as follows: fold change = 2–Δ(ΔCT), where ΔCT = CT target − CT housekeeping, and Δ(ΔCT) = ΔCT treated − ΔCT control.

### 2.4. Western Blot Analysis

The liver and pancreas tissue samples were frozen at −80 °C until homogenization (Politron Generator FSH-G 5/085 from Thermo Fisher Scientific, Waltham, MA, USA,) in extraction buffer (100 mg/mL) on ice. Lysis buffer (20 mm Tris–HCl, pH 7.5, 1 mM EDTA, 150 mM NaCl, 0.1% SDS, 1% Igepal, 30 mM sodium pyrophosphate, 50 mM sodium fluoride, 1 mM sodium orthovanadate) and a protease inhibitor cocktail (Sigma-Aldrich) at a concentration of 4 μL/mL were employed. Homogenates were centrifuged for 15 min at 15,000 rpm and 4 °C. The concentration of the proteins in each homogenate was measured by the bicinchoninic acid (BCA) protein assay (Thermo Fisher Scientific, Waltham, Massachusetts, USA). Blots were visualized using a chemiluminescence (ECL) detection kit Western blotting substrate (Fisher Scientific, Madrid, Spain). Signals were captured by the ChemiDoc XRS and Imaging System (Bio-rad, Richmond, CA, USA). The density of bands was measured by version 2.0.1 of the Image Lab Software (Bio-rad, Richmond, CA, USA).

The employed antibodies were: anti-β-tubulin (1:1000, ab6046 from Abcam, Cambridge, UK); anti-PGC-1α (1:500, sc-518025 from Santa Cruz Biotechnology, Dallas, TX, USA); anti-p65 (1:1000, #8242 from Cell Signaling Technology, Danvers, MA, USA); anti-phospho-p65 (Ser 536) (1:100, #3033 from Cell Signaling); anti-Nitro-tyrosine (1:1000, #9691 from Cell Signaling); anti-STAT3 (1:1000, #9132 from Cell Signaling); anti-phospho-STAT3 (Tyr705) (1:1000, #9131 from Cell Signaling); anti-GAPDH (1:1000, #2118 from Cell Signaling); anti-NOS2 (1:1000, ab178945 from Abcam); anti-IgG (1:1000, #7076 from Cell Signaling).

### 2.5. Co-Immunoprecipitation

Protein–protein interactions were analyzed by co-immunoprecipitation experiments. Whole-cell extracts were prepared and subjected to immunoprecipitation with specific antibodies against PGC-1α (sc-518025, Santa Cruz, Dallas, TX, USA) and p65 (1:1000, #8242 from Cell Signalling) as previously described [25]. The presence of both NF-κB and p-STAT3 in immunoprecipitates was evaluated by a Western blot with their corresponding antibodies (p65 and p-STAT3).

### 2.6. Redox Pairs and Protein Nitration and Chlorination by UPLC-MS/MS Analysis

Redox pairs, namely, oxidized glutathione (GSSG)/reduced glutathione (GSH), γ-glutamilcystine/γ-glutamilcysteine and cysteine (Cyss)/cysteine (Cys), were analyzed from the frozen liver samples homogenized in phosphate buffered saline (PBS) with 10 mM N-ethylmaleimide. Then, perchloric acid was added to obtain a 4% concentration and centrifuged at 15,000× *g* for 15 min at 4 °C. The concentration of analytes was determined in the supernatants by Ultra Performance Liquid Chromatography—mass spectrometry UPLC-MS/MS. This method was performed following the protocol of Escobar et al. [26]

Protein nitration and chlorination were determined by calculating the ratio 3NO2-Tyrosine/p-Tyrosine and 3Cl-Tyrosine/p-Tyrosine. The protocol consists of homogenizing frozen liver with lysis buffer (100 mg/mL). Next proteins were precipitated with trichloroacetic acid (TCA) (10%, *v*/*v*), and pellets were resuspended in sodium acetate (50 mmol/L, Ph 7.2) (Sigma-Aldrich, St. Louis, MO, USA). Immediately, the protein digestion from tissue extracts was carried out according to Hensley’s method [27]. To finish pronase activity, TCA was used to precipitate it. Then, samples were centrifuged (5000 rpm, 4 °C, 5 min) and the supernatant from each sample was injected into the chromatographic system to be quantified by UPLC-MS/MS according to Torres-Cuevas et al. [28].

Data were acquired and processed with the MassLynx 4.1 software and the QuanLynx 4.1 software (Waters), respectively.

### 2.7. Energy Charge Determined by UPLC-MS/MS

The energy charge (E.C.) is an index relative to ATP, ADP and AMP concentrations as indicated by the formula E.C. = ((ATP) + 0.5(ADP))/((ATP) + (ADP) + (AMP)) [29].

The determination has been performed in the liver tissue by means of ultra-high-resolution liquid chromatography coupled to a mass spectrometry tandem (UPLC-MS/MS). The system used is Acquity UPLC-Xevo TQD from Waters (Milford, MA, USA). The samples were processed from frozen liver samples (−80 °C), homogenized in water: methanol (1:3) cold at 4 °C (100 mg/mL). In this step, the methanol precipitates the proteins present in the homogenate and, once precipitated, to be eliminated, the samples were centrifuged for 20 min at 15,000 rpm at 4 °C. The supernatant obtained was analyzed by UPLC-MS/MS according to Jiang Y et al. with slight modifications [30]. The precipitate was suspended in ammonium acetate buffer pH = 7 to determine the protein concentration in the sample.

### 2.8. Biochemical Assays

Lipase, amylase and aspartate aminotransferase (AST) activity were determined in plasma by the LIPASE-LQ, AMYLASE-LQ and GOT (AST)-LQ, respectively, (Spinreact, Girona, Spain). The procedures were performed according to the indications of the kit.

Triglycerides and total lipids were determined in liver tissue by the TRIGLYCERIDES-LQ and TOTAL LIPIDS, respectively (Spinreact, Girona, Spain). The procedures were performed according to the indications of the kit in liver homogenate in PBS (100 mg/mL). Results were normalized by protein concentration.

### 2.9. Histological Analysis

Pieces of pancreas were rapidly removed, fixed in 4% paraformaldehyde for 24 h and embedded in paraffin, 4 µM sections were prepared using an automatic microtome, and then stained with hematoxylin and eosin for microscopic analysis. Pancreatic sections were assessed at 20× magnification over 10 separate fields for severity of pancreatitis by scoring for edema and inflammatory infiltrate according to Van Laethem et al. [31].

### 2.10. Statistical Analysis

All the values were expressed as means ± SE. To analyze the significance of the quantitative variables, the statistical treatment one-way analysis of variance (ANOVA), followed by Tukey’s post-hoc test, was used. *p* < 0.05 was the limit to accept statistically significant differences. The results were managed with the statistical tool of the GraphPad Prism 8 software (GraphPad Software Inc., La Jolla, CA, USA).

## 3. Results

### 3.1. PGC-1α Levels are Up-Regulated in Mice Livers after Inducing Acute Pancreatitis

In order to determine the role of PGC-1α in liver tissue during AP, our first approach was to measure its transcriptional and protein levels. First, we confirmed the appropriate AP induction by a histological analysis of the pancreas and determined plasma amylase and lipase activity (see Figure S1 Supplementary Materials).

**Figure 1:**
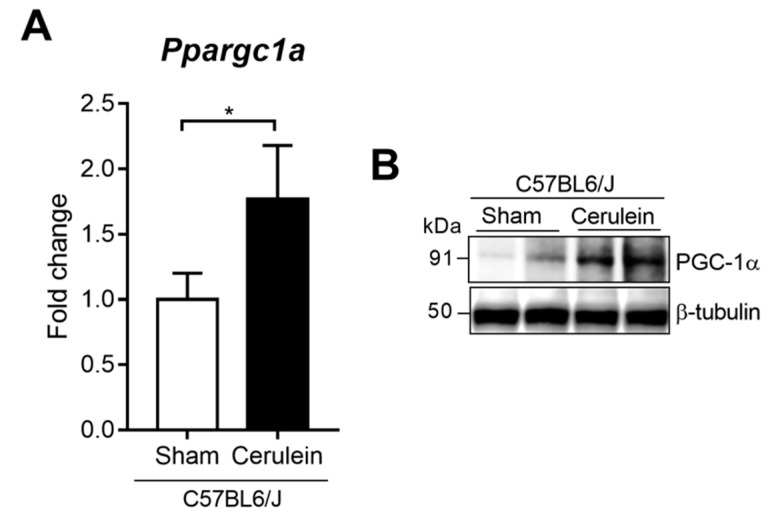
(**A**) mRNA relative expression of *Ppargc1a* versus *Tbp* (TATA-binding protein; housekeeping) in the livers of the control (Sham) and 1 h after the cerulein-induced acute pancreatitis (Cerulein) mice. (**B**) Representative Western blot of PGC-1α in the livers of the control (Sham) and 1 h after the cerulein-induced acute pancreatitis (Cerulein) mice. β-tubulin was used as the loading control. There were six mice per group. Statistical difference is indicated as * *p* < 0.05 vs. Sham.

Interestingly, *Ppargc1a* mRNA expression was up-regulated in the livers of the mice with AP (Figure 1A). Then, we set out to verify whether this transcriptional up-regulation would result in a rise in protein levels. The Western blot analysis showed that PGC-1α protein expression was higher in the livers of the mice with cerulein-induced pancreatitis than in the control mice (Figure 1B).

### 3.2. PGC-1α Restrains Nos2 Expression in the Liver after Acute Pancreatitis in Mice

**Figure 2:**
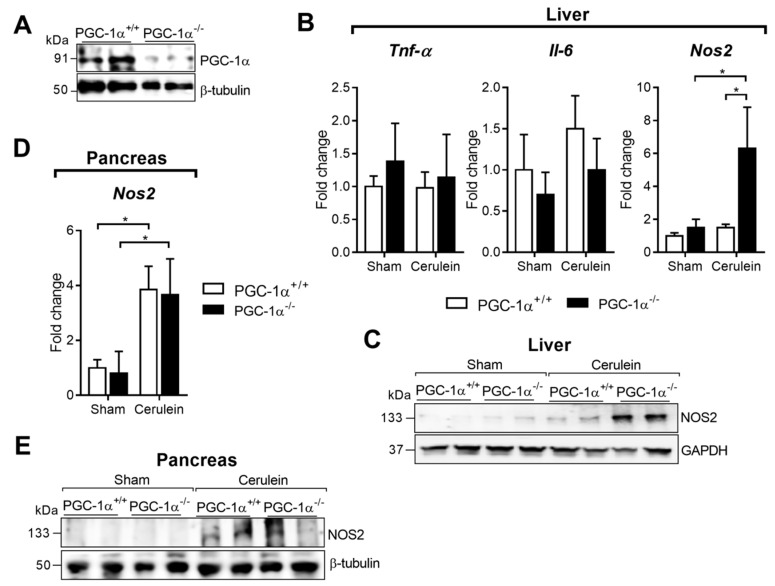
(**A**) Representative Western blot of PGC-1α in the livers of PGC-1α^+/+^ (WT—wild-type) and PGC-1α^-/-^ (KO—knockout) mice. Β-tubulin was used as the loading control. (**B**) mRNA relative expression of *Tnfa*, *Il6* and *Nos2* versus *Tbp* (TATA binding protein; housekeeping) in the livers of the sham PGC-1α^+/+^ (WT) and PGC-1α^-/-^ (KO) mice and at 1 h after cerulein-induced acute pancreatitis (AP) (Cerulein). (**C**) Representative Western blot of NOS2 in the livers of the sham PGC-1α^+/+^ (WT) and PGC-1α^-/-^ (KO) mice and at 1 h after cerulein-induced AP (Cerulein). GAPDH was used as the loading control. (**D**) mRNA relative levels of *Nos2* versus *Tbp* in the pancreas of the sham PGC-1α^+/+^ (WT) and PGC-1α^-/-^ (KO) mice and at 1 h after cerulein-induced AP (Cerulein). (**E**) Representative Western blot of NOS2 in the pancreas of the sham PGC-1α^+/+^ (WT) and PGC-1α^-/-^ (KO) mice and at 1 h after cerulein-induced AP (Cerulein). Β-tubulin was used as the loading control. There were six mice per group. Statistical difference is indicated as *p* * < 0.05 vs. Sham.

Having determined the induction of PGC-1α in the liver during AP, the inflammatory response of AP in liver tissue was studied using the PGC-1α deficient mice (Figure 2A).

For this purpose, the hepatic transcriptional expression of cytokines *Tnfα* and interleukin-6 (*Il6*), as well as *Nos2*, a marker of cellular stress, was analyzed. The *Tnfα* and *Il6* mRNA levels did not vary after administering cerulein to any experimental group (Figure 2B). *Nos2* gene expression remained unchanged in the wild-type (WT) mice after inducing AP (Figure 2B). However, and unlike the changes observed in the *Tnfα* and *Il6* gene expressions, a marked increase (around 6-fold) was detected in the transcription of *Nos2* in the livers of the PGC-1α deficient mice with AP (Figure 2B). Western blot also showed that NOS2 protein expression was higher in the livers of the KO mice with AP (Figure 2C).

We previously reported that *Il6* mRNA levels were selectively up-regulated in the pancreas of the PGC-1α KO mice with AP compared to their WT littermates, conversely to other pro-inflammatory cytokines [32]. Here, we observed that although *Nos2* mRNA expression was up-regulated in the pancreas upon pancreatitis induction, no significant differences appeared between the PGC-1α KO mice and the WT mice (Figure 2D). Similarly, the Western blot also showed NOS2 induction during AP, although there was no change between WT and KO mice with pancreatitis (Figure 2D).

### 3.3. PGC-1α Avoids the Assembly of the Complex between p65 and Phospho-STAT3 in the Liver during Experimental Acute Pancreatitis

By taking into account our previous studies, in which the plasma levels of IL-6 increased in the PGC-1α KO mice with pancreatitis [32], we considered studying whether NF-κB and STAT3 activation are involved in inducing *Nos2* in the liver upon PGC-1α deficiency.

**Figure 3:**
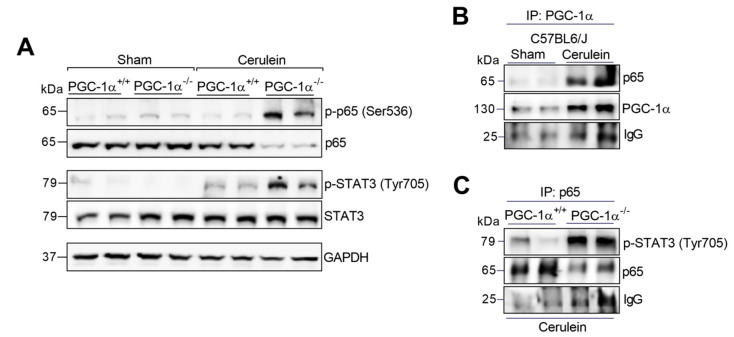
(**A**) Representative Western blot of p-p65 (Ser536), p65, p-STAT3 (Tyr705) and STAT3 in the livers of the sham PGC-1α^+/+^ (WT) and PGC-1α^-/-^ (KO) mice and at 1 h after cerulein-induced AP (Cerulein). GAPDH was used as the loading control. (**B**) Representative Western blot of p65 and PGC-1α in the PGC-1α immunoprecipitate of the livers of the sham PGC-1α^+/+^ (WT) mice and at 1 h after cerulein-induced AP mice. (**C**) Representative Western blot of p-STAT3 (Tyr705) and p65 in the p65 immunoprecipitate of the livers of the PGC-1α^+/+^ (WT) mice and PGC-1α^-/-^ (KO) mice with pancreatitis (Cerulein). IgG was used as the loading control. There were six mice per group.

The Western blot analysis showed a dramatic increase in the phosphorylation of the p65 subunit of NF-κB in the livers of the PGC-1α KO mice after AP induction (Figure 3A). Regarding STAT3 activation, AP triggered an increase in its phosphorylated form in both the WT and KO mice in liver tissue (Figure 3A). Interestingly, the STAT3 phosphorylation levels were higher in the PGC-1α-deficient mice compared to the WT under AP conditions (Figure 3A).

It has been previously shown that the p65 subunit of NF-κB may complex with p-STAT3 favoring the expression of *Nos2* [33]. Additionally, our research group has shown that PGC-1α forms a protein complex with p65 in the pancreas during AP [32]. Based on this background, we decided to evaluate the role of PGC1alpha in p65-binding to p-STAT3 in the liver during AP. According to our immunoprecipitation studies, the augmented levels of PGC-1α found in the liver after the induction of AP bound to p65 (Figure 3B) as we previously reported in pancreatic tissue. However, the lack of PGC-1α markedly increased the binding of p65 to p-STAT3 in KO mice with pancreatitis (Figure 3C), suggesting that the induction of PGC-1α in the liver during AP may inhibit the formation of the p65/p-STAT3 complex.

### 3.4. PGC-1α Deficiency Downregulates Antioxidant Gene Expression and Increases Oxidative Stress in the Liver with Acute Pancreatitis

**Figure 4:**
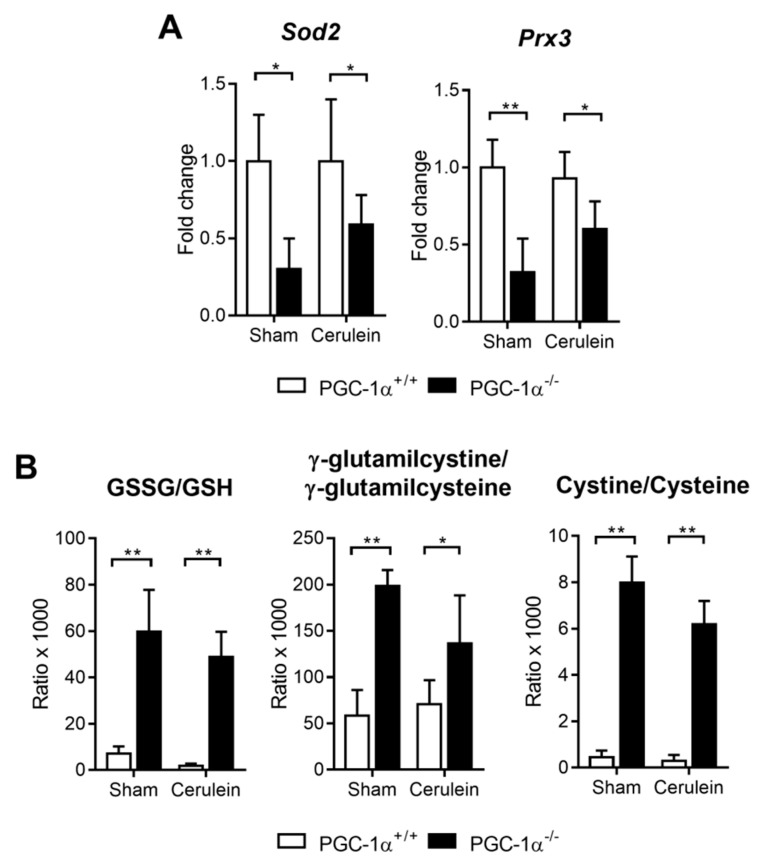
(**A**) mRNA relative expression of *Sod2* and *Prx3* versus the *Tbp* (TATA-binding protein; housekeeping) in the livers of the sham PGC-1α^+/+^ (WT) and PGC-1α^-/-^ (KO) mice and at 1 h after cerulein-induced AP (Cerulein). (**B**) The GSSG/GSH, g-glutamilcystine/g-glutamilcysteine and cystine/cysteine ratios in the livers of the sham PGC-1α^+/+^ (WT) and PGC-1α^-/-^ (KO) mice and at 1 h after cerulein-induced AP (Cerulein). There were six mice per group. The statistical difference is indicated as * *p* < 0.05 and ** *p* < 0.001.

Taking into account the role of PGC-1α in the regulation of antioxidant genes, the mRNA expression of *Sod2* and *Prdx3* was measured in the liver of the PGC-1α KO and WT mice. As expected, PGC-1α deficiency triggered the down-regulation of *Sod2* and *Prdx3* under basal conditions and during pancreatitis (Figure 4A).

Additionally, the liver redox status was assessed by measuring disulfide pairs, Cyss/Cys, γ-glutamyl cystine/γ-glutamyl cysteine and GSSG/GSH. According to the antioxidant genes expression, the GSSG/GSH, γ-glutamyl cystine/γ-glutamyl cysteine and Cyss/Cys ratios were significantly higher in the livers of the PGC-1α KO mice than in the WT mice (Figure 4B), which supports the relevance of PGC-1α for maintaining redox homeostasis in liver during AP.

### 3.5. PGC-1α Prevents Protein Nitration in the Liver after Inducing Experimental Acute Pancreatitis

**Figure 5:**
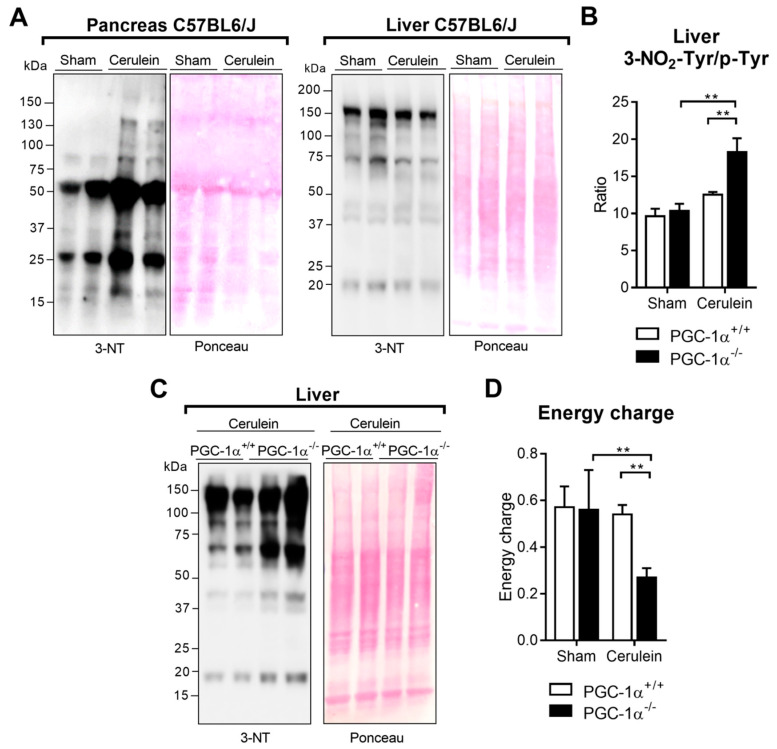
(**A**) Representative Western blot of 3-Nitrotyrosine (NT) in the pancreas and liver of the control (Sham) and 1 h after cerulein-induced acute pancreatitis (Cerulein) mice. Ponceau was used as the loading control. (**B**) Determination of the 3NO2-Tyr/p-Tyr ratio in the livers of the sham PGC-1α^+/+^ (WT) and PGC-1α^-/-^ (KO) mice and at 1 h after cerulein-induced AP (Cerulein). (**C**) Representative Western blot of 3-NT in the liver after inducing acute pancreatitis (Cerulein) in the PGC-1α^+/+^ (WT) and PGC-1α^-/-^ (KO) mice. Ponceau was used as the loading control. (**D**) Energy charge in the livers of the sham PGC-1α^+/+^ (WT) and PGC-1α^-/-^ (KO) mice and at 1 h after cerulein-induced AP (Cerulein). There were six mice per group. The statistical difference is indicated as ** *p* < 0.001.

In accordance with the *Nos2* mRNA levels found in the pancreas and liver of the mice with AP, we observed that AP produced increased protein nitration in the pancreas, but not in the liver of the WT mice (Figure 5A). Remarkably, and consistently with the increased *Nos2* mRNA expression observed in the livers of the PGC-1α KO mice with pancreatitis, we detected higher levels of 3-nitrotyrosine, a marker of protein nitration measured by mass spectrometry, in the livers of these mice compared to the other groups (Figure 5B). This increase in 3-nitrotyrosine was confirmed by Western blot in the livers of the PGC-1α KO mice with cerulein-induced pancreatitis (Figure 5C). However, we did not observe changes in 3-chlorotyrosine, parameter associated with inflammation (see Figure S2A in the Supplementary Materials) [34].

Considering that mitochondria are the main source of peroxynitrite and are also highly susceptible to the effects of nitrosative stress [35], we set out to calculate the energy charge in liver tissue during AP. The results revealed a significant drop in this parameter in the livers of the KO mice with pancreatitis (Figure 5D), although these changes did not alter the serum AST levels in AP (see Figure S2B in the Supplementary Materials).

### 3.6. PGC-1α Levels Lower in the Livers of Obese Mice under Basal Conditions and during Pancreatitis

PGC-1α has been previously reported to be dysregulated in obese animals and patients [18]. As we know that obesity increases the risk of systemic complications in AP [36], we decided to measure the PGC-1α levels in the livers of the lean and obese mice under basal conditions and during AP. First, we corroborated that high-fat diet induced weight gain, hyperglycemia and fatty liver (Table 3).

**Figure 6:**
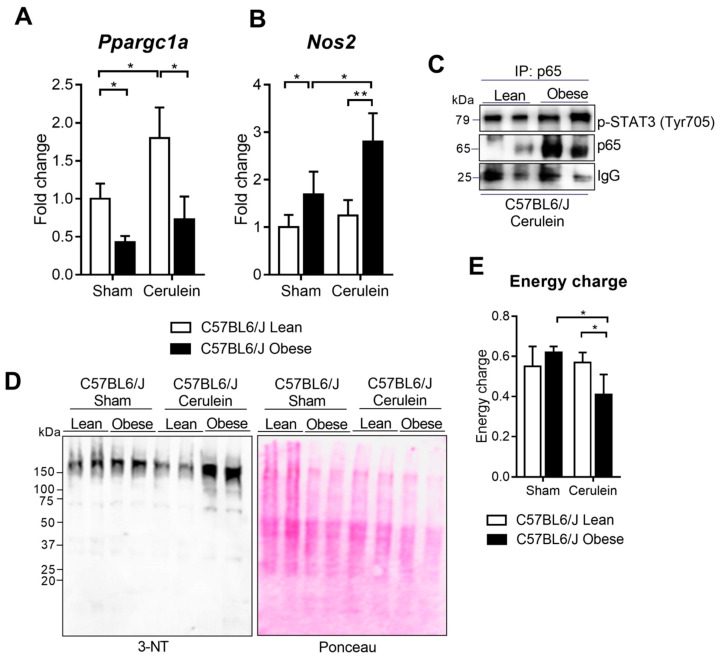
(**A**) mRNA relative expression of *Ppargc1a* versus *Tbp* (TATA-binding protein; housekeeping) in the livers of the sham lean and obese mice and 1 h after cerulein-induced acute pancreatitis (Cerulein). (**B**) mRNA relative expression of *Nos2* versus *Tbp* in the livers of the sham lean and obese mice and 1 h after cerulein-induced acute pancreatitis (Cerulein). (**C**) Representative Western blot of p-STAT3 (Tyr705) and p65 in p65 immunoprecipitate of the sham lean and obese mice and 1 h after cerulein-induced acute pancreatitis (Cerulein). IgG was used as the loading control. (**D**) Representative Western blot of 3-NT in the livers of the sham lean and obese mice and 1 h after cerulein-induced acute pancreatitis (Cerulein). Ponceau was used as the loading control. (**E**) Energy charge in the livers of the sham lean and obese mice and 1 h after cerulein-induced acute pancreatitis (Cerulein). There were five mice per group. Statistical difference is indicated as * *p* < 0.05 and ** *p* < 0.001.

The *Ppargc1a* mRNA levels were markedly down-regulated in the livers of the obese mice under basal conditions (Figure 6A). Interestingly, the obese mice did not exhibit any increased *Ppargc1a* expression, which was found in the lean mice after inducing AP (Figure 6A).

After bearing in mind our findings in the PGC-1α KO mice, we decided to study whether the PGC-1α deficiency detected in the obese mice would impact nitrosative stress in the livers of these mice during AP. We observed that *Nos2* gene expression was up-regulated in the obese mice under basal conditions (Figure 6B). We stress that this up-regulation was higher in the obese mice with pancreatitis (Figure 6B). We also observed a marked increase in the binding of p65 to p-STAT3 in the obese mice with pancreatitis (Figure 6C), which supports the notion that lack of PGC-1α in obese mice livers may be associated with inducing *Nos2* during AP.

The protein nitration levels were higher in the livers of the obese mice than those of the lean mice with pancreatitis (Figure 6D). Interestingly, the obese mice showed a lower hepatic energy charge in relation to the lean mice with pancreatitis (Figure 6E). As we observed in the PGC-1α-deficient mice, this drop in energy charge did not change the AST levels in serum (see Figure S2C in the Supplementary Materials).

## 4. Discussion

Of the different systemic complications to occur during AP, the commonest are those that affect the cardiovascular, renal, and pulmonary systems [14]. Although most of the pancreatic enzymes and mediators released by an inflamed pancreas pass through the liver before entering systemic circulation, liver failure is a rare pathophysiological condition in the course of AP with a significant prognostic value for its severity [15,16]. It is noteworthy that obesity, which is a risk factor for AP development, decisively contributes to the appearance of systemic complications, including those that affect liver function [13,16]. The present work highlights the role of PGC-1α in regulating the relation between obesity and liver injury in the course of AP. We particularly demonstrate that obesity impairs hepatic PGC-1α up-regulation and, thus, enhances *Nos2* transcriptional expression and causes nitrosative stress in the liver during AP.

Our results show that AP induction in mice increases transcriptional and protein PGC-1α expressions in the liver, which is crucial for preventing *Nos2* up-regulation. In pancreatic tissue, our group has recently shown that PGC-1α binds to phospho-p65 to repress *Il6* gene expression during pancreatitis. Consequently, PGC-1α deficiency enhances NF-κB-dependent *Il6* transcription in the pancreas by augmenting circulating IL-6 levels and increasing local and systemic inflammatory responses [32]. In accordance with these findings, here we found higher levels for both NF-κB and STAT3 activation in the livers of the PGC-1α KO mice with pancreatitis. PGC-1α deficiency may be responsible for activating p65, as we have previously reported in pancreatic tissue [32]. It is also accompanied by STAT3 hyperactivation, probably due to the high IL-6 plasma levels achieved in PGC-1α KO mice during pancreatitis. Yet despite the activation of both the NF-κB and STAT3 signaling pathways in the livers of the PGC-1α KO mice, we did not observe any increase in the *Tnfα* or *Il6* expression levels in these mice. Strikingly, and unlike pancreatic tissue, lack of PGC-1α in the liver specifically induced *Nos2* transcription during AP development.

Although *Nos2* expression can be induced by different transcription factors (AP-1, C/EBP, CREB, IRF-1, NF-κB, NF-IL6, Oct-1, SRF, STAT1α) [37], its transcription has also been described to be specifically regulated by the formation of complexes between p65 and STAT3 in the promoter region of *Nos2* [33]. During pancreatitis, our results revealed that p65 bound to PGC-1α in the liver. The formation of this complex has been previously reported in both human cardiac cells and the heart, liver and pancreas of mice [32,38,39]. The present work shows that, as a consequence of PGC-1α deficiency, p65 bound to p-STAT3 in the liver, which could justify the particular increase in the *Nos2* transcription found in the livers of the PGC-1α KO mice.

The present results confirm the presence of nitrosative stress in the pancreas during AP in accordance with augmented pancreatic *Nos2* expression levels [8,9]. Nevertheless, we did not note any increase in the protein nitration levels in the liver after inducing pancreatitis, which is consistent with the PGC-1α-dependent repression of *Nos2* in this tissue. It is well-known that nitrosative stress is implied in the liver pathophysiology [40]. In particular, peroxynitrite accumulation in LPS-treated mice is one of the commonest causes for acute liver injury [41,42]. In fact, hepatocytes respond to LPS treatment with IL-6- and TNF-α-mediated NO production, which induces a hepatic acute phase response [43]. Furthermore, reactive nitrogen species (RNS) production contributes to nitrate critical amino acid residues, which render hepatocytes more susceptible to oxidative damage [40]. In our work, lack of PGC-1α in the liver lowered the antioxidant defense gene expression, which triggered increased oxidative stress. This pro-oxidant environment in the livers of the PGC-1α KO mice, together with specific *Nos2* induction, would explain the more marked nitration pattern observed in the livers of these mice with pancreatitis. Hence, these results demonstrate that PGC-1α is crucial for preventing nitrosative stress in the liver during AP development.

The mitochondrion is the organelle most susceptible to the consequences of nitrosative stress [35]. Numerous studies have shown that mitochondrial dysfunction participates both in the progression of organ failure and in the development of the systemic inflammatory response syndrome (SIRS) [44]. Interestingly, Trumbecakaite et al. showed that AP triggered mitochondrial failure in the pancreas, kidney, and lung, while the liver preserved mitochondrial function during the development of PA [45]. According to this work, our results reveal that the energy charge was unchanged in the liver of WT mice with pancreatitis. However, the induction of PA in mice deficient in PGC-1α caused a decrease in the energy charge in the liver of these mice, confirming the essential role of PGC-1α in the maintenance of mitochondrial homeostasis during inflammatory processes.

According to our results, *Nos2* transcription in the liver during pancreatitis seems to depend on PGC-1α levels. PGC-1α expression lowers in skeletal muscle in both mice with genetic obesity (ob/ob) and fat diet-induced obesity [46]. Furthermore, it has been found that a high-fat diet inhibits PGC-1α expression in mice liver and induces non-alcoholic fatty liver development [39]. Accordingly, our research group recently showed a drop in the protein and transcriptional levels of PGC-1α in the pancreas of obese Zucker rats and in mice with fat diet-induced obesity [32]. Here, we confirm that a high-fat diet in mice down-regulated *Ppargc1a* expression in the liver under basal conditions, and that this decrease was also maintained after inducing AP. Consequently, we observed higher *Nos2* gene expression and protein nitration levels and decreased energy charge in the livers of obese mice compared to the lean mice with pancreatitis.

Obesity is associated with increased *Nos2* expression in insulin-sensitive tissue in rodents and humans [47]. Abdominal obesity increases free fatty acids in the liver, which induces superoxide anion formation and up-regulates *Nos2* gene expression to result in peroxynitrite synthesis and to lead to both mitochondrial dysfunction and liver injury [48]. After taking into account that high-fat diet administration aggravates AP-induced hepatic injury via oxidative stress [49], our herein reported findings provide new insights into the detrimental effect of obesity on liver complications during AP, which highlights the key role of PGC-1α deficiency in this regard.

## 5. Conclusions

The present work demonstrates the essential role of PGC-1α in inflammatory response and nitrosative stress during AP, particularly in liver tissue. First, pancreatitis leads to marked PGC-1α induction in the liver. Our results also suggest that PGC-1α binds to p65 by acting as a repressor of NF-κB transcriptional activity and, in turn, prevents the formation of a transcriptional complex between p65 and p-STAT3. Therefore, PGC-1α deficiency triggers the increase in the *Nos2* expression mediated by the p65/p-STAT3 complex in the liver, and consequently results in higher protein nitration levels and a decrease in the energy charge. Finally, obesity triggers PGC-1α deficiency in the liver and enhances nitrosative stress during pancreatitis. Therefore, our results highlight the protective role of PGC-1α in the liver for preventing nitrosative stress during AP.

## Figures and Tables

**Table 1:** The oligonucleotides used for RT-qPCR.

Target Gene (mm)	Direct/Reverse Oligonucleotide
*Ppargc1a* (Gene ID: 19017)	F --> TTAAAGTTCATGGGGCAAGC R --> TAGGAATGGCTGAAGGGATG
*Sod2* (Gene ID: 20656)	F --> GGCCAAGGGAGATGTTACAA R --> GAACCTTGGACTCCCACAGA
*Prx3* (Gene ID: 11757)	F --> CAAGAAAGAATGGTGGTTTGG R --> TGCTTGACGACACCATTAGG
*Nos2* (Gene ID: 18126)	F -->GCATCCCAAGTACGAGTGGGT R -->GAAGTCTCGAACTCCAATC
*Tbp* (Gene ID: 21374)	F -->CAGCCTTCCACCTTATGCTC R --> CCGTAAGGCATCATTGGACT

**Table 2:** The TaqMan^®^ probe used for RT-qPCR.

Target Gene (mm)	Sonda TaqMan
*Tnfα* (Gene ID: 21926)	Mm00443258_g1
*Il6* (Gene ID: 16193)	Mm00446190_m1
*Tbp* (Gene ID: 21374)	Mm01277042_m1

**Table 3:** Parameters of high-fat diet-induced obese mice.

	Lean Group	Obese Group
**Body weight (g)**	22.9 ± 1.1	29.7 ± 1.8 **
**Blood glucose (mg/dl)**	137.8 ± 25.8	201.6 ± 50.3 *
**Liver Triglycerides (mg/g protein)**	86.4 ± 22.4	138.8 ± 28.4 **
**Liver Total Lipids (mg/g protein)**	665.8 ± 208.4	1581.7 ± 437.7 **

The statistical difference is indicated as * *p* < 0.05 and ** *p* < 0.001.

## Supplementary Materials

The following are available online at https://www.mdpi.com/2076-3921/9/9/887/s1, Figure S1 and Figure S2 are in the attached supplementary document.
