# Supplementary material for: Impairment of PGC-1 Alpha Up-Regulation Enhances Nitrosative Stress in the Liver during Acute Pancreatitis in Obese Mice
Source: Antioxidants (Basel). 2020 Sep 19;9(9):887. doi: 10.3390/antiox9090887 (PMC7554866; doi:10.3390/antiox9090887)
Supplement: Supplementary file 1 [file antioxidants-09-00887-s001.pdf]

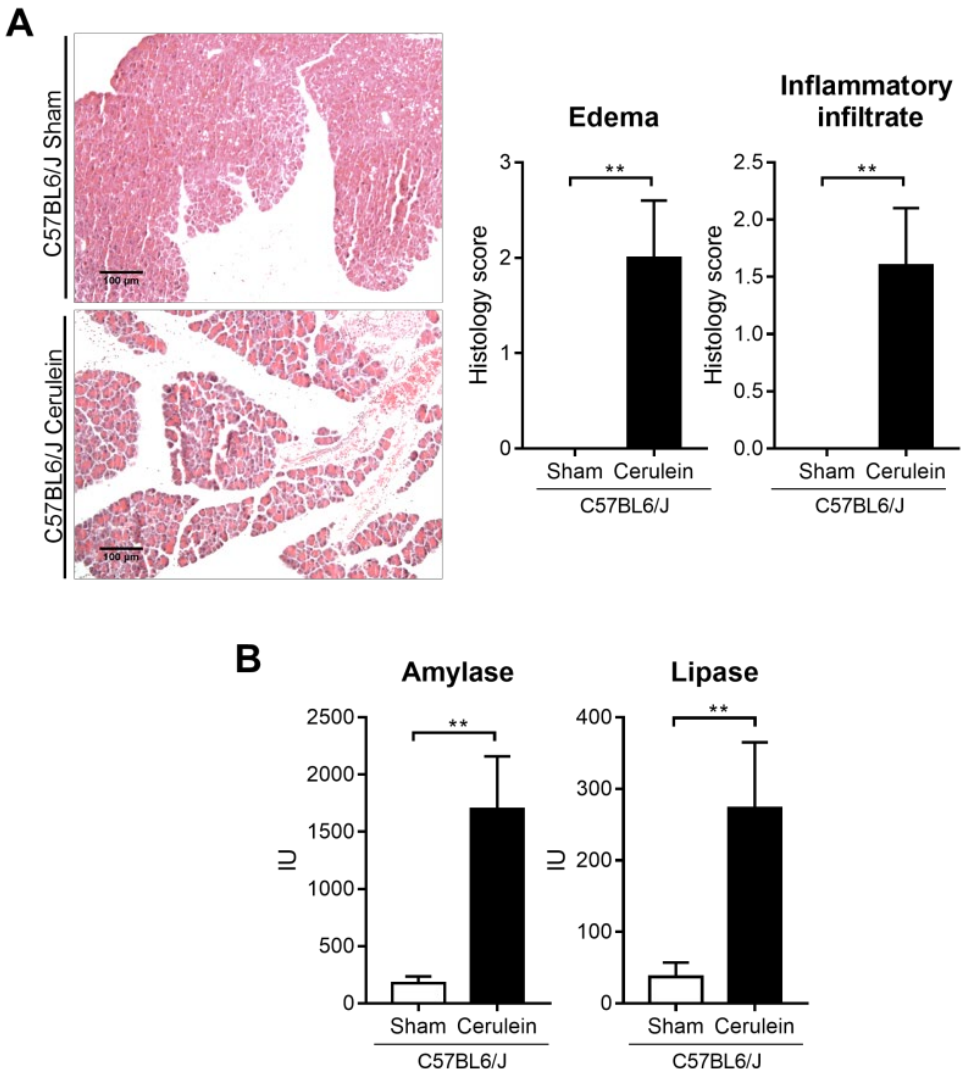

Figure S1. (**A**) Representative histology and histology score for edema and inflammatory infiltrate in the pancreas of the control (Sham) and 1 h after the cerulein-induced acute pancreatitis (Cerulein) mice. (**B**) Pancreatic amylase and lipase activity in plasma of the control (Sham) and 1 h after the cerulein-induced acute pancreatitis (Cerulein) mice. There were six mice per group. Statistical difference is indicated as ** $p < 0.01$ vs. Sham.

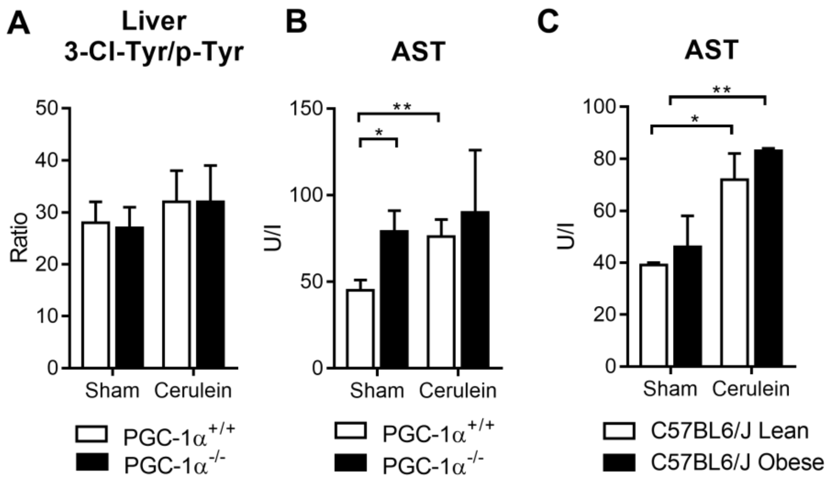

Figure S2. (**A**) Determination of the 3-Chloro-Tyr/p-Tyr ratio in the livers of the sham PGC-1$\alpha^{+/+}$ (WT) and PGC-1$\alpha^{-/-}$ (KO) mice and at 1 h after cerulein-induced AP (Cerulein). (**B**) Aminotransferase (AST) activity in plasma of the sham PGC-1$\alpha^{+/+}$ (WT) and PGC-1$\alpha^{-/-}$ (KO) mice and at 1 h after cerulein-induced AP (Cerulein). (**C**) AST activity in plasma of the sham lean and obese mice and 1 h after cerulein-induced acute pancreatitis (Cerulein). There were between five and six mice per group. The statistical difference is indicated as * $p < 0.05$ and ** $p < 0.001$.
